# Differences in Lead Content and Macronutrients in *Undaria pinnatifida* Edible Seaweed at Harvest and Compositional Changes During Blanching and Salting

**DOI:** 10.3390/foods15213998

**Published:** 2026-11-07

**Authors:** Jiaqi Chang, Chi Zhang, Yihan Liu, Yingying Guo, Han Zhang, Yichao Ma, Dandan Ren, Qiukuan Wang, Shu Liu, Yunhai He

**Affiliations:** 1 College of Food Science and Engineering, Dalian Ocean University, Dalian 116023, China; changjiaqi020326@163.com (J.C.); chigelaile@163.com (C.Z.); lyh8mh@163.com (Y.L.); zhanghan@dlou.edu.cn (H.Z.); 18640876812@163.com (Y.M.); rdd@dlou.edu.cn (D.R.); wqk320@dlou.edu.cn (Q.W.); 2 Key Laboratory of Aquatic Product Processing and Utilization of Liaoning Province, Dalian Ocean University, Dalian 116023, China; 3 National R&D Branch Center for Seaweed Processing, Dalian Ocean University, Dalian 116023, China; 4 Yellow Sea Fishery Research Institute, Chinese Academy of Fishery Sciences, Qingdao 266071, China; guoyy@ysfri.ac.cn

**Keywords:** harvest period, lead (Pb) accumulation, salting, *Undaria pinnatifida*, nutritional components, principal component analysis

## Abstract

As dietary preferences diversify, aquatic foods remain a global staple, providing high-quality protein, essential fatty acids, and other essential nutrients. However, maintaining the quality and safety of seafood remains challenging. Salting is the primary postharvest processing method for the brown seaweed *Undaria pinnatifida* and can affect product quality and lead (Pb) accumulation. This study investigated changes in nutritional components, bioactive compounds, and Pb content throughout the harvest season and subsequent blanching and salting. Relationships among these variables were examined to identify suitable salting conditions. Samples were collected at nine 7-day intervals from the coastal waters of Dalian, China, and analyzed at the fresh, blanched, and salted stages. The maximum protein and alginate contents in fresh samples were 21.95% and 36.60% (dry weight), respectively, with a corresponding Pb content of 0.235 mg/kg. Principal component and Pearson correlation analyses showed that Pb accumulation was associated with protein and alginate contents. Salting with 40% salt for 48 h preserved favorable texture and color while maintaining Pb content below 0.35 mg/kg. These findings indicate that both harvest stage and processing affect nutritional quality and Pb levels. Although salting promotes Pb accumulation, controlling salt concentration and salting duration can limit this effect while preserving product quality. Salting with 40% salt for 48 h is therefore suggested as a suitable condition for producing salted *U. pinnatifida* with desirable texture, color, and Pb content. These findings provide a scientific basis for improving the quality and safety of salted brown algal products.

## 1. Introduction

Aquatic foods are important sources of high-quality protein, essential fatty acids, and other nutrients for populations worldwide. However, their safety and quality may be affected by contaminants originating from production environments or introduced during processing and storage. Understanding the sources and behavior of contaminants throughout the food production chain is therefore essential for effective risk control and for ensuring the quality and safety of aquatic food products.

*Undaria pinnatifida* is an economically important brown seaweed widely cultivated in China. It contains alginate, mannitol, fucoidan, iodine, and other bioactive constituents, supporting its potential applications in the food and nutraceutical industries [1]. Salting is widely used for postharvest preservation of *U. pinnatifida* because of its simplicity, low cost, and ability to extend shelf life [2]. Dalian, at the southern tip of the Liaodong Peninsula between the Yellow Sea and the Bohai Sea, offers favorable conditions for seaweed cultivation. The region has a long history of aquaculture and an established industry encompassing seedling production, offshore cultivation, processing, and marketing [3]. Major *U. pinnatifida* cultivation areas include the Jinshitan and Lüshunkou coastal waters, making Dalian an important production region for this species in China.

Despite the economic importance of *U. pinnatifida*, expanding aquaculture and coastal industrial development may place additional pressure on the marine environment. Heavy metal contamination is a particular concern because seaweeds accumulate metals from seawater, potentially transferring them into the food chain [4]. Postharvest processing may also affect nutritional quality and contaminant concentrations. Blanching and salting can alter seaweed composition, while processing materials and conditions may influence contaminant levels and their distribution within the product. Evaluating the safety of salted *U. pinnatifida* therefore requires consideration of metal accumulation during cultivation and changes associated with processing.

Lead (Pb) is a food safety concern because exposure may adversely affect human health, particularly with prolonged dietary intake [5]. Despite efforts to protect Dalian’s marine environment, Pb contamination remains relevant to coastal food production. Previous studies have documented heavy metal bioaccumulation in *U. pinnatifida* during cultivation and changes in metal contents during food processing. However, most studies have focused on total heavy metal concentrations in seaweed products. Interactions between Pb and major intrinsic components, particularly proteins and polysaccharides such as alginate and fucoidan, remain insufficiently understood. The relative contributions of cultivation-related bioaccumulation and postharvest salting to final Pb levels also remain difficult to quantify. Furthermore, the effects of salting conditions on Pb concentration and chemical forms within algal tissues have not been fully elucidated. This study used *U. pinnatifida* harvested from Dalian’s coastal waters to investigate changes in nutritional composition and Pb content during salting. Different salting conditions were evaluated, and Pb chemical forms were analyzed across processing stages to explore potential migration pathways. The findings are expected to support processing optimization and improve quality and safety control for salted brown algal foods.

## 2. Materials and Methods

### 2.1. Experimental Reagents

Potassium sulfate (AR, ≥99.0%), copper(II) sulfate pentahydrate (AR, ≥99.0%), sodium hydroxide (AR, ≥96.0%), boric acid (AR, ≥99.5%), silver nitrate (AR, ≥99.8%), calcium acetate (AR, ≥99.0%), sodium thiosulfate (AR, ≥99.0%), potassium iodide (AR, ≥99.5%), periodic acid (ACS reagent, ≥99.0%), and anhydrous diethyl ether (AR, ≥99.0%) were purchased from Macklin Biochemical Co., Ltd. (Shanghai, China). Nitric acid (AR, 65.0–68.0%), sulfuric acid (AR, 95.0–98.0%), and hydrochloric acid (AR, 36.0–38.0%) were purchased from Sinopharm Chemical Reagent Co., Ltd. (Shanghai, China).

Potassium sulfate, copper(II) sulfate pentahydrate, sodium hydroxide, boric acid, sulfuric acid, and hydrochloric acid were used to determine protein content by the Kjeldahl method. Silver nitrate and calcium acetate were used to measure alginate content. Periodic acid, sodium thiosulfate, and potassium iodide were used for the mannitol assay. Anhydrous diethyl ether was used for lipid determination by Soxhlet extraction. Nitric acid was used for sample digestion before ICP-OES analysis.

### 2.2. Sample Collection and Pretreatment

Fresh *Undaria pinnatifida* samples were collected from the Jinshitan coastal waters of Dalian (39°05′ N, 122°02′ E), Yellow Sea, China. The samples were taxonomically identified by Professor Zeyu Zhang of Dalian Ocean University. Sampling was conducted at nine 7-day intervals from mid-to-late February to early May 2025, covering the entire harvest season. At each time point, healthy individuals of uniform size were randomly selected. Selected individuals had intact thalli, blades without signs of decay, and no visible surface contaminants. Blanched samples were prepared by immersing fresh algae in heated seawater, followed by immediate cooling. Salted samples were prepared by treating blanched algae with 45% salt (*w*/*w*) for 48 h. Alternatively, blanched and salted *U. pinnatifida* samples were supplied by Jiahai Food Co., Ltd, Dalian, China. For each batch, 30 individuals of each sample type (fresh, blanched, and salted) were randomly selected and placed in insulated containers with ice packs. Samples were transported to the laboratory within 4 h. On arrival, the stipes and blades were separated, and the blades were retained for compositional analysis. All samples were stored at −20 °C until analysis. Harvest dates and sample categories are listed in Table A1.

### 2.3. Determination of Major Components in Undaria pinnatifida

**Figure 1:**
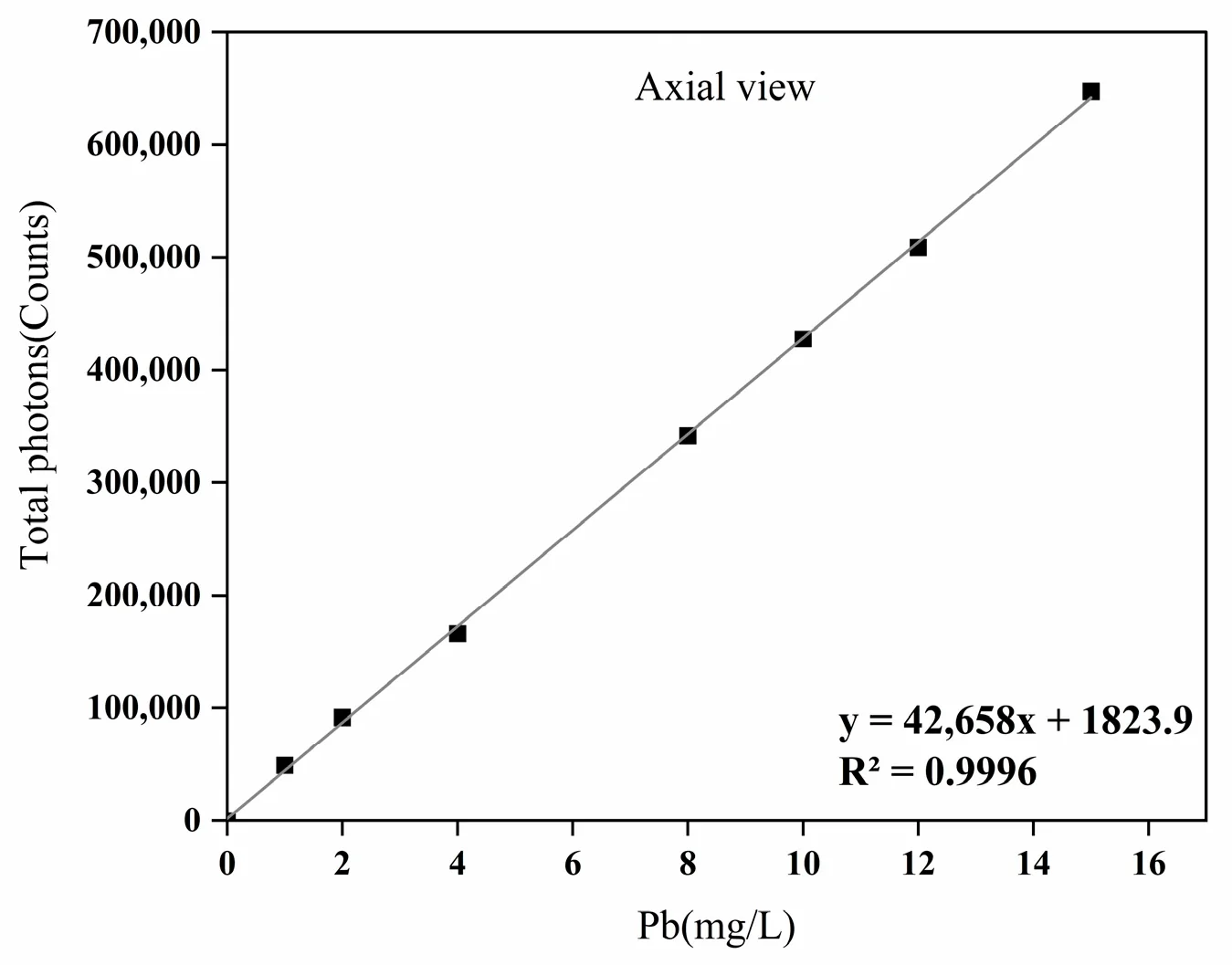
Axial-view calibration curve for Pb determination.

The following procedures were used to determine the major components of *U. pinnatifida*. Moisture content was measured by direct oven drying. Weighing bottles were pre-dried at 101–105 °C to constant mass. Approximately 2 g of sample, weighed to 0.0001 g, was placed in each bottle and dried at 101–105 °C. Samples were cooled in a desiccator and repeatedly dried to constant mass. Moisture content was calculated from mass loss [6]. Fat content was determined by Soxhlet extraction. Samples were placed in cellulose thimbles and extracted with petroleum ether for 6–10 h at a reflux rate of 6–8 cycles per hour. The solvent was then evaporated, and the receiving flasks were dried at 100 °C to constant mass, cooled in a desiccator, and weighed. Fat content was calculated from the mass difference [7]. For ash determination, samples were heated on an electric hot plate over low heat until fully carbonized and no smoke remained. They were then incinerated in a muffle furnace (SX2-4-10,YETUO Technologies Inc., Shanghai, China) at 550 °C ± 25 °C for 4 h, cooled to approximately 200 °C, and weighed [8]. Protein content was determined by the Kjeldahl method. Approximately 0.25 g of sample, weighed to 0.0001 g, was transferred to a digestion tube. Potassium sulfate (6 g) and copper sulfate pentahydrate (0.4 g) were added as catalysts, followed by digestion with concentrated sulfuric acid at 380–420 °C. After cooling, the samples were distilled, and the released ammonia was absorbed in boric acid solution and quantified by titration [9]. Mannitol was determined by its rapid oxidation to aldehydes by periodic acid, whereas sugars and polysaccharides reacted more slowly. Mannitol was indirectly quantified by iodometric titration with sodium thiosulfate [10]. Alginate content was determined by calcium acetate titration. Pretreated alginic acid samples were suspended in distilled water, mixed with 0.1 mol/L calcium acetate solution, and stirred thoroughly. The mixtures were maintained at room temperature for 2 h to complete the replacement reaction. The liberated acetic acid was titrated with 0.1 mol/L sodium hydroxide solution using phenolphthalein as the indicator. A blank was analyzed in parallel, and alginate content was calculated from the volume of NaOH consumed. Iodine content was determined after sample carbonization and ashing. Iodide was oxidized to iodate in an acidic medium using liquid bromine. The iodate then oxidized potassium iodide to release iodine, which was titrated with sodium thiosulfate using starch as the indicator [11]. For Pb analysis, solid samples (1.0 g) or liquid samples (5 mL) were mixed with 5 mL of nitric acid (AR, 65–68%, Pb ≤ 0.00001%). The mixtures were allowed to stand for 1 h and then digested in microwave digestion vessels. Digestion conditions were adapted from Monajjemi [12] to suit the sample characteristics (Table 1). Digestates were analyzed by inductively coupled plasma optical emission spectrometry (ICP-OES; Optima 8000, PerkinElmer Inc., Waltham, MA, USA) in axial-view mode. Instrumental settings were as follows: high-purity argon pressure, 0.8 MPa; nitrogen pressure, 0.6 MPa; power, 1300 W; plasma gas flow, 12 L/min; auxiliary gas flow, 0.2 L/min; nebulizer gas flow, 0.55 L/min; and analytical pump flow rate, 1.5 mL/min. Spike-recovery experiments were performed to evaluate the accuracy of Pb determination. Appropriate amounts of Pb standard solution were added to *U. pinnatifida* samples before microwave digestion. Pb recoveries ranged from 93.2% to 107.5%, within the acceptable range for trace element analysis. The limit of detection (LOD) was 0.001 mg·L^−1^, and the limit of quantification (LOQ) was 0.003 mg·L^−1^ for this digestion method. The calibration curve for measuring Pb content under this equipment parameter is shown in (Figure 1).Results were expressed on a dry-weight or fresh-weight basis, as appropriate. Pb content was expressed in mg·kg^−1^ for solid samples and mg·L^−1^ for liquid samples.

Fucoidan-enriched fractions were extracted from dried *U. pinnatifida* powder according to He [13] with minor modifications, and we used 30% ethanol solution to enhance the removal of alginate to some extent, facilitating the precipitation of crude polysaccharides. Dried samples were ground into a fine powder and mixed with distilled water at a solid-to-liquid ratio of 1:15 (*w*/*v*). The suspensions were hydrolyzed using a composite enzyme preparation to release cell wall-associated polysaccharides. After enzymatic treatment, aqueous extraction was performed, and the resulting supernatants were collected and precipitated twice with ethanol. The precipitates were collected and freeze-dried to obtain crude fucoidan-enriched fractions. Total sugar content was determined by the phenol–sulfuric acid colorimetric method using D-glucose as the reference standard [13].

### 2.4. Optimization of Salting Conditions for the Processing of Blanched Undaria pinnatifida

Blanched *U. pinnatifida* from a single homogeneous production batch was randomly divided into five groups, each prepared in triplicate, to optimize salting conditions. Surface sediments and impurities were removed by thorough rinsing with clean water. Sodium chloride was added at 30%, 35%, 40%, 45%, and 50% (*w*/*w*) of the fresh algal weight [2]. Salt and algae were layered alternately in salting vessels, and a weighted lid was placed on top. The released brine completely submerged the samples throughout salting at ambient temperature. Subsamples were collected from each group at 24, 36, and 48 h [14]. Moisture, fat, ash, protein, and Pb contents were determined using the methods described above.

### 2.5. Texture Profile Analysis of Salted Undaria pinnatifida

Texture was analyzed using a TMS-Pro texture analyzer (Food Technology Inc., Sterling, VA, USA). The instrument has interchangeable load cells, a maximum load of ±2500 N, and a travel stroke of 320 mm. Its position resolution is 2.5 μm, and its test speed ranges from 1 to 1000 mm/min. The instrument supports multiple test modes, including texture profile analysis (TPA), with texture parameters calculated automatically using TL-Pro software (v1.8). Samples were cut into uniform 3 cm × 3 cm squares. Residual surface salt crystals were gently removed before the samples were centered on the test platform. TPA was performed using a TA/50 probe with the following settings: load cell capacity, 1000 N; trigger force, 1.5 N; target deformation, 30% of the original sample height; compression speed, 60 mm/min; and probe rise height after the first compression, 10 mm. Thus, the probe returned to 10 mm above the sample surface before the second compression cycle [15]. Six replicate pieces were independently tested for each treatment. Hardness, springiness, cohesiveness, and chewiness were calculated from the resulting force–time curves.

### 2.6. Color Measurement of Salted Undaria pinnatifida

Color was measured using a CR-400 chroma meter (Konica Minolta Sensing Inc., Sakai, Osaka, Japan). This portable instrument uses diffuse illumination/0° viewing geometry and an 8 mm measurement aperture. It is equipped with a pulsed xenon lamp, completes measurements within 1 s, and provides high repeatability (ΔE*ab ≤ 0.07). CIE L*a*b* color parameters were recorded for *U. pinnatifida* blades subjected to different salt concentrations and salting durations. Surface salt particles were removed before measurements were taken at three locations on each sample. Each sample was measured in triplicate [16].

### 2.7. Extraction and Speciation of Chemical Forms of Pb in Undaria pinnatifida

Pb chemical forms in *U. pinnatifida* were determined by sequential extraction according to Zhang [17] and Zhao [18,19]. Fresh, blanched, and salted samples were sequentially extracted with 80% ethanol (*v*/*v*), deionized water, 1 mol/L NaCl, 2% acetic acid (*v*/*v*), and 0.6 mol/L HCl. For each extraction, frozen tissues were homogenized with the extractant at a ratio of 1:50 (*w*/*v*), shaken at 25 °C for 15 h, and centrifuged at 8000× *g* for 10 min. The supernatants were collected, and the precipitates were re-extracted twice with the same extractant under identical conditions for 2 h each, followed by centrifugation. The supernatants from all three extractions were combined to obtain the corresponding fraction (F1–F5). The final residual precipitate was collected as the sixth fraction (F6). All fractions were microwave-digested, and Pb content was determined by ICP-OES as described above.

### 2.8. Health Risk Assessment Indices

Four health risk assessment indices were calculated to evaluate the dietary safety of salted *Undaria pinnatifida*: daily intake of metals (DIM), hazard quotient (HQ), cancer risk (CR), and total cancer risk (TCR).

The daily intake of metals (DIM) was calculated as follows:

DIM = (C × IR × EF × ED)/(BW × AT)

C: Pb concentration in salted *U. pinnatifida* (mg/kg);

IR: daily seaweed ingestion rate (kg/d);

EF: exposure frequency (d/year);

ED: exposure duration (years);

BW: body weight (kg);

AT: averaging time (d).

Noncarcinogenic risk was expressed as the hazard quotient (HQ), calculated as follows:

HQ = DIM/RfD

RfD: reference dose for Pb. An HQ < 1 indicates an acceptable noncarcinogenic risk.

Cancer risk (CR) and total cancer risk (TCR) were calculated as follows:

CR = DIM × SF

TCR = ΣCR

SF: cancer slope factor for Pb. Generally, CR < 10^−6^ indicates negligible carcinogenic risk, whereas 10^−6^ < CR < 10^−4^ indicates acceptable carcinogenic risk.

Exposure parameters for children, adolescents, and adults were selected from published food safety studies on seaweed products. The Pb concentration (C) of the optimized salted *U. pinnatifida* was set at 0.35 mg/kg. Ingestion rate (IR), exposure duration (ED), and body weight (BW) were specified separately for each population group. Exposure frequency (EF) was 365 d/year. For noncarcinogenic risk assessment, averaging time (AT) was calculated as ED multiplied by 365 days. For carcinogenic risk assessment, AT was fixed at 70 × 365 days to represent lifetime exposure. The Pb reference dose (RfD) and cancer slope factor (SF) were obtained from the United States Environmental Protection Agency (USEPA), Washington, DC, USA. Because only Pb was evaluated, TCR was equal to CR.

### 2.9. Statistical Analysis

Data are expressed as the mean ± standard deviation (SD) of three independent replicates. Statistical analyses were performed using SPSS 16.0 (SPSS Inc., Chicago, IL, USA). Figures were generated using Origin 2024 (OriginLab Corporation, Northampton, MA, USA). A *p* value < 0.05 was considered statistically significant.

## 3. Results and Discussion

### 3.1. Harvest-Time and Blanching Effects on Thallus Morphology of Undaria pinnatifida

The stipe weight proportion and morphological parameters of *U. pinnatifida* varied over the harvest period (Table 2). In fresh samples, the stipe weight proportion increased progressively from 33.76% in early February to 43.99% in late April. This indicates a significant increase in the proportion of supporting tissue as the thalli aged during the later cultivation stages. After blanching, the stipe weight proportion generally increased, reaching a maximum of 49.19% in late March. These findings suggest that blanching altered thallus morphology primarily through partial water loss and tissue contraction, while preserving the overall increase with harvest time.

Fresh thallus length increased from 132 cm in early February to 146 cm in late April and remained relatively stable from mid-to-late March onward. This pattern reflects favorable environmental conditions and sustained algal growth in the cultivation area. Fresh thallus width peaked at 46 cm in mid-to-late March and remained relatively high from mid-March to mid-April. This period was characterized by vigorous morphological development and substantial biomass accumulation. These findings agree with previous reports of pronounced seasonal variation in biomass accumulation rates of farmed *U. pinnatifida* in different coastal waters [20,21].

Blanching notably reduced thallus length at all harvest stages. This reduction was attributed to heat-induced tissue contraction and structural densification. The effect on thallus width varied with harvest stage. Width decreased notably after blanching early in the season, whereas blanched samples had comparable or greater widths than fresh samples later in the season. This response may reflect age-related changes in cell wall structure, water-holding capacity, and tissue swelling during thallus senescence. Solana [2] reported that blanching significantly affected *U. pinnatifida* texture and that mild heating (≤75 °C for ≤120 s) effectively preserved desirable textural properties. Blanching may therefore be more influential than subsequent salting in determining final product quality.

### 3.2. Changes in the Major Components of Fresh U. pinnatifida During the Harvesting Period

**Figure 2:**
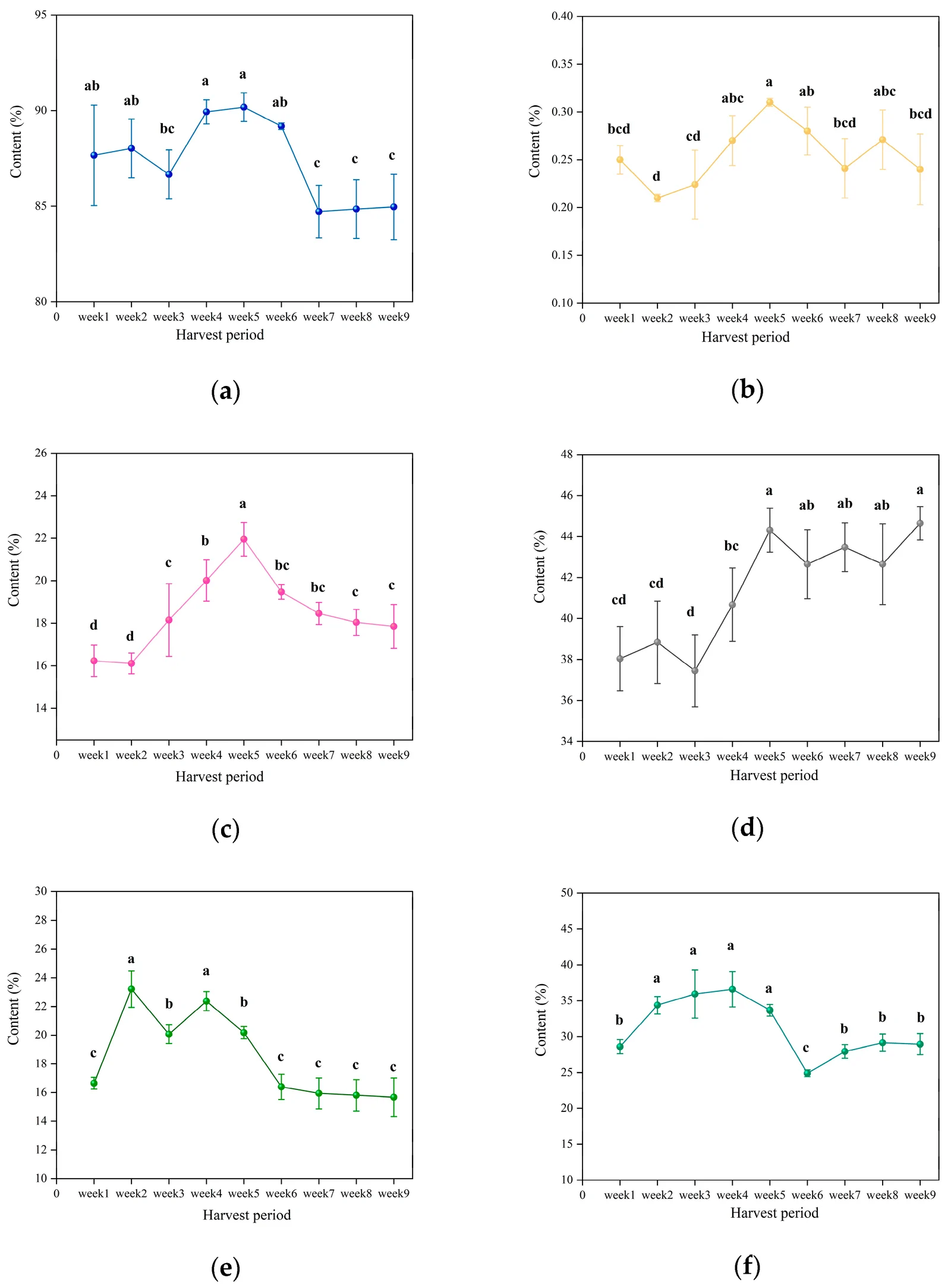

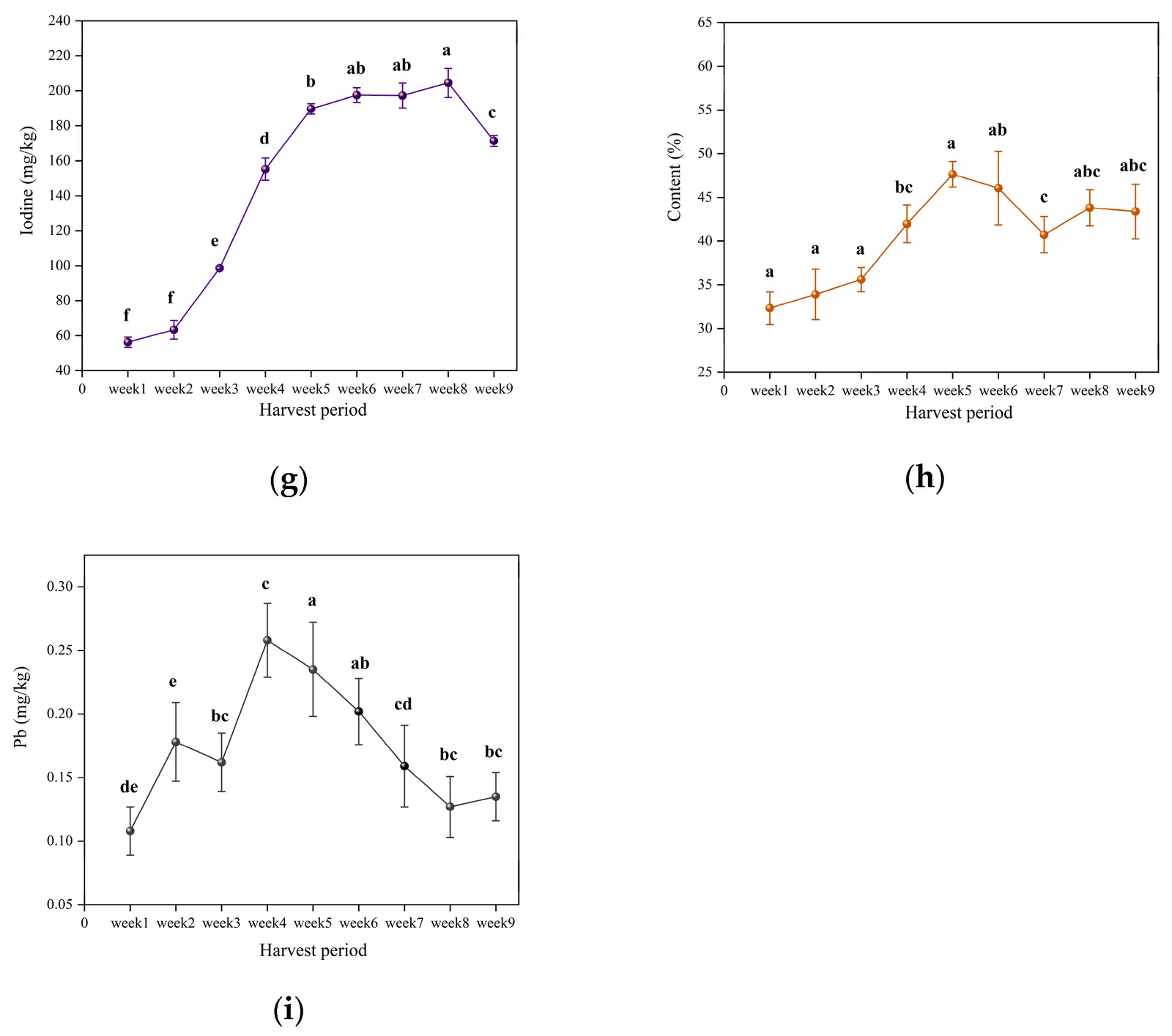
Changes in the major components of fresh *U. pinnatifida* during the harvest period: (**a**) moisture; (**b**) fat; (**c**) protein; (**d**) ash; (**e**) mannitol; (**f**) alginate; (**g**) iodine; (**h**) fucoidan; and (**i**) Pb. All contents except moisture are expressed on a dry-weight basis. Letters (a, b, c, d, e, f) in the figure/table represent the results of multiple-comparison analysis while groups with completely different letters indicate significant differences at *p* < 0.05, and do not directly represent the magnitude of numerical values.

Fresh *U. pinnatifida* exhibited distinct seasonal changes in composition during the harvest period (Figure 2). Moisture content remained above 85%, decreasing slightly during the middle and late stages (Figure 2a). This indicates that the fresh algae maintained high freshness and quality during this period. Fat content remained below 0.31% throughout the harvest period (Figure 2b), confirming that *U. pinnatifida* is a low-fat, low-calorie food. Gerasimenko [22] likewise reported higher total fat contents at the seedling and juvenile stages than at maturity, with relatively stable levels thereafter. Protein content increased from late February, peaked at approximately 21.95% in mid-to-late March, and decreased slightly toward late April (Figure 2c). This decline may reflect reduced protein synthesis during late maturation and protein consumption during spore formation and reproduction. Similar seasonal patterns have been reported in other macroalgae. Clélia reported that the protein content of *Agarophyton vermiculophyllum* reached 21.2% in March and declined to 17.1% in April [23]. Forbord found that protein content in cultivated *Saccharina latissima* initially increased and then declined during cultivation [24]. Ash content increased markedly from March onward, reaching 44.65% at the end of the harvest season (Figure 2d). This increase was likely associated with blade expansion during maturation, which facilitated mineral uptake from seawater. Marco similarly linked algal mineral accumulation to changes in thallus morphology and growth [25].

Mannitol content peaked at 23.21% in early March and then declined gradually (Figure 2e). As a functional sugar alcohol and temporary storage compound, mannitol may be progressively consumed to support growth and reproduction during late maturation, resembling the protein trend. Klindukh reported that mannitol in *Fucus vesiculosus* accumulated in spring, was consumed during summer and autumn, and reached its lowest level in winter [26].

Alginate content followed a seasonal trend similar to that of protein (Figure 2f). It increased from early March to a maximum of 36.60% in mid-to-late March and then declined. As a major cell wall component, alginate is actively synthesized during early blade expansion but may subsequently be mobilized to support reproductive structure formation. Apoya reported higher alginate contents in *U. pinnatifida* blades than in sporophylls from Japanese waters, with higher contents before sporophyll development [27].

Iodine content generally increased during the harvest period, reaching a maximum of 204.55 mg/kg (Figure 2g), similarly to the trend in ash content. Udo reported higher iodine contents in several brown algae from Irish waters in winter than in summer [28]. That study suggested that iodine retention depends on environmental iodine concentrations. The differing trends may reflect variation in algal species and local marine conditions.

The extraction yield of fucoidan sulfate from fresh *U. pinnatifida* was approximately 1.02% on a dry-weight basis. Total sugar content in the extracted fucoidan initially increased and then remained relatively stable during the harvest period (Figure 2h). It peaked at 47.64% from late March to early April, significantly exceeding the level at the beginning of the season. Mak also reported a gradual increase in fucoidan sulfate content in *U. pinnatifida* harvested from New Zealand waters [29].

Pb content was positively correlated with protein and alginate contents and peaked at 0.258 mg/kg in mid-to-late March (Figure 2i). Davis reported strong cadmium adsorption by alginate extracted from *Sargassum* [30], while Weiyan suggested that increased organic acid content could delay Pb adsorption equilibrium in algae [31]. To investigate the source of Pb during processing, Pb concentration in the cultivation seawater was measured and found to be below 0.5 μg/L. Despite this low environmental concentration, *U. pinnatifida* has a strong capacity to absorb and accumulate Pb from seawater. This bioaccumulation may pose a non-negligible risk of Pb contamination in the final product.

### 3.3. Effects of Blanching on the Major Components of Undaria pinnatifida

Blanching, the first step in salted *U. pinnatifida* processing, involved immersing fresh algae in seawater at 80–95 °C for 40–50 s. The algae were then immediately cooled with seawater until their temperature approached that of the cooling medium. This treatment produced a glossy, uniformly bright-green appearance and a moderately soft texture.

**Figure 3:**
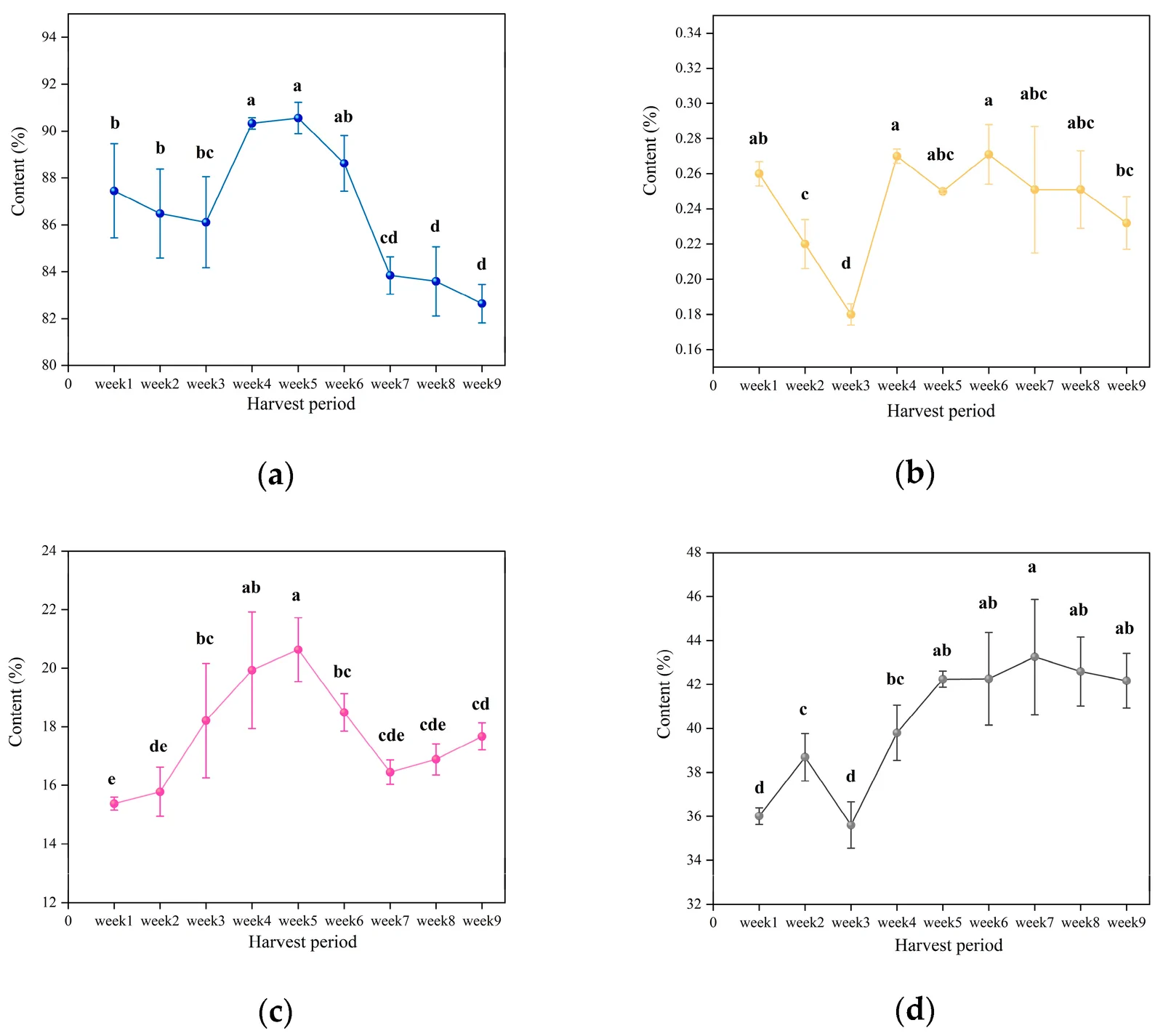

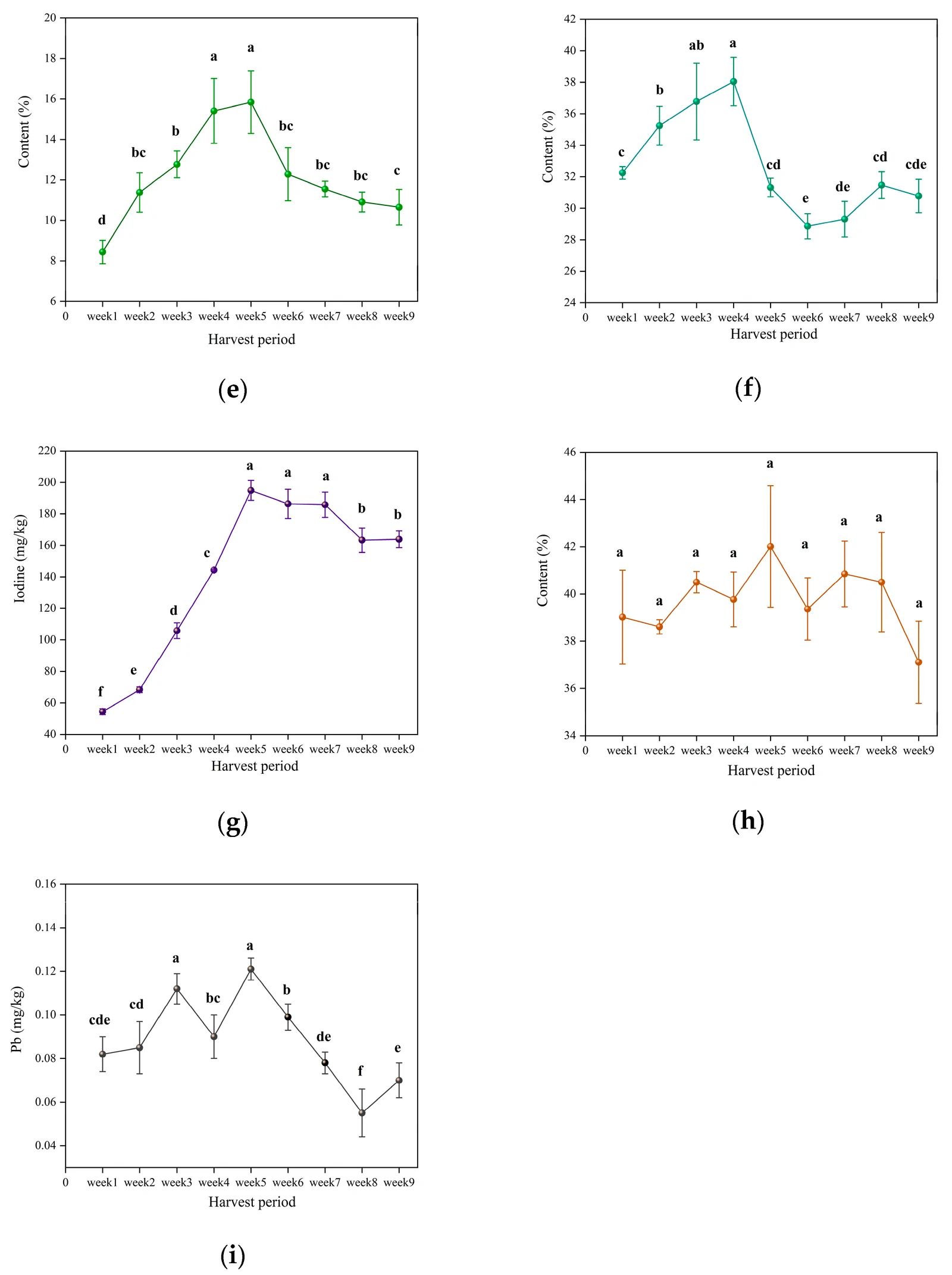
Changes in the major components of blanched *U. pinnatifida* during the harvest period: (**a**) moisture; (**b**) fat; (**c**) protein; (**d**) ash; (**e**) mannitol; (**f**) alginate; (**g**) iodine; (**h**) fucoidan; and (**i**) Pb. All contents except moisture are expressed on a dry-weight basis. Letters (a, b, c, d, e, f) in the figure/table represent the results of multiple-comparison analysis while groups with completely different letters indicate significant differences at *p* < 0.05, and do not directly represent the magnitude of numerical values.

The composition of blanched samples generally followed the seasonal trends observed in fresh thalli, with some component-specific differences (Figure 3). Blanching did not substantially affect moisture, fat, or iodine contents (Figure 3a,b,g). Fat content remained low, and iodine content was relatively stable. Protein and ash contents decreased slightly, by up to approximately 1.32% and 2.07%, respectively (Figure 3c,d).

Mannitol is a small, highly water-soluble molecule abundant in algal cells and was substantially leached during rapid hot-water treatment and cooling. Mannitol losses after blanching ranged from 4.12% to 11.38% relative to fresh samples (Figure 3e). Hamid similarly reported that blanching markedly reduced most low-molecular-weight metabolites without significantly affecting amino acid or sugar composition [14].

Measured alginate content was slightly higher in blanched than in fresh samples, peaking at 38.05% in mid-to-late March (Figure 3f). This apparent increase may reflect blanching-induced changes in cellular structure that facilitated alginate extraction or conversion during analysis.

Iodine content decreased slightly after blanching, reaching a maximum of 194.90 mg/kg (Figure 3g). The extraction yield of fucoidan sulfate was approximately 1.12%, slightly higher than that of fresh samples. Its total sugar content was somewhat lower, peaking at 42.07% (Figure 3h).

Pb content was consistently lower in blanched samples than in corresponding fresh samples throughout the harvest period (Figure 3i). The highest values were 0.121 mg/kg in blanched samples and 0.235 mg/kg in fresh samples. This reduction suggests that blanching removes some physically adsorbed or weakly bound Pb from algal surfaces and tissues. Afiah reported that precooking removed 56% of Pb from the red alga *Gracilaria*, supporting the role of heat treatment in reducing heavy metals [32]. Blanching wastewater contained (0.16 ± 0.02)% protein, (1.04 ± 0.24)% mannitol, and (1.58 ± 0.62)% alginate, whereas Pb was undetectable. This may be because the blanching process takes place in a container of extremely large volume; under these conditions, the change in lead content is below the LOD of ICP-OES, so no significant increase in lead content was detected in the wastewater. These findings indicate that blanching causes losses of some nutritional components while measurably reducing Pb content, potentially improving product safety.

### 3.4. Effects of Salting on the Major Components of Undaria pinnatifida

Salting inhibits microbial growth, extends shelf life, and imparts a characteristic flavor to *U. pinnatifida* products. Industrial salting typically uses 35–45% salt (*w*/*w*), relative to algal weight, for approximately 48 h. This process also induces further changes in composition.

**Figure 4:**
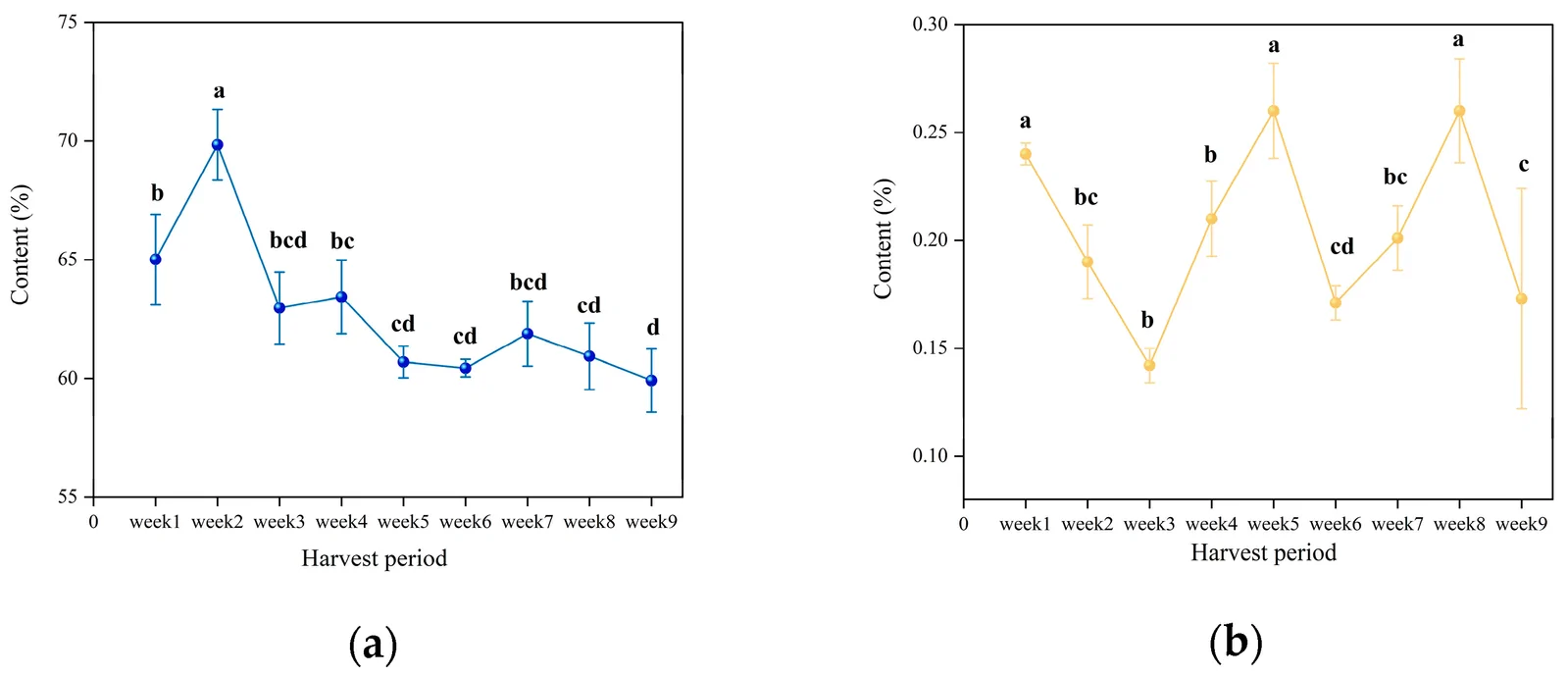

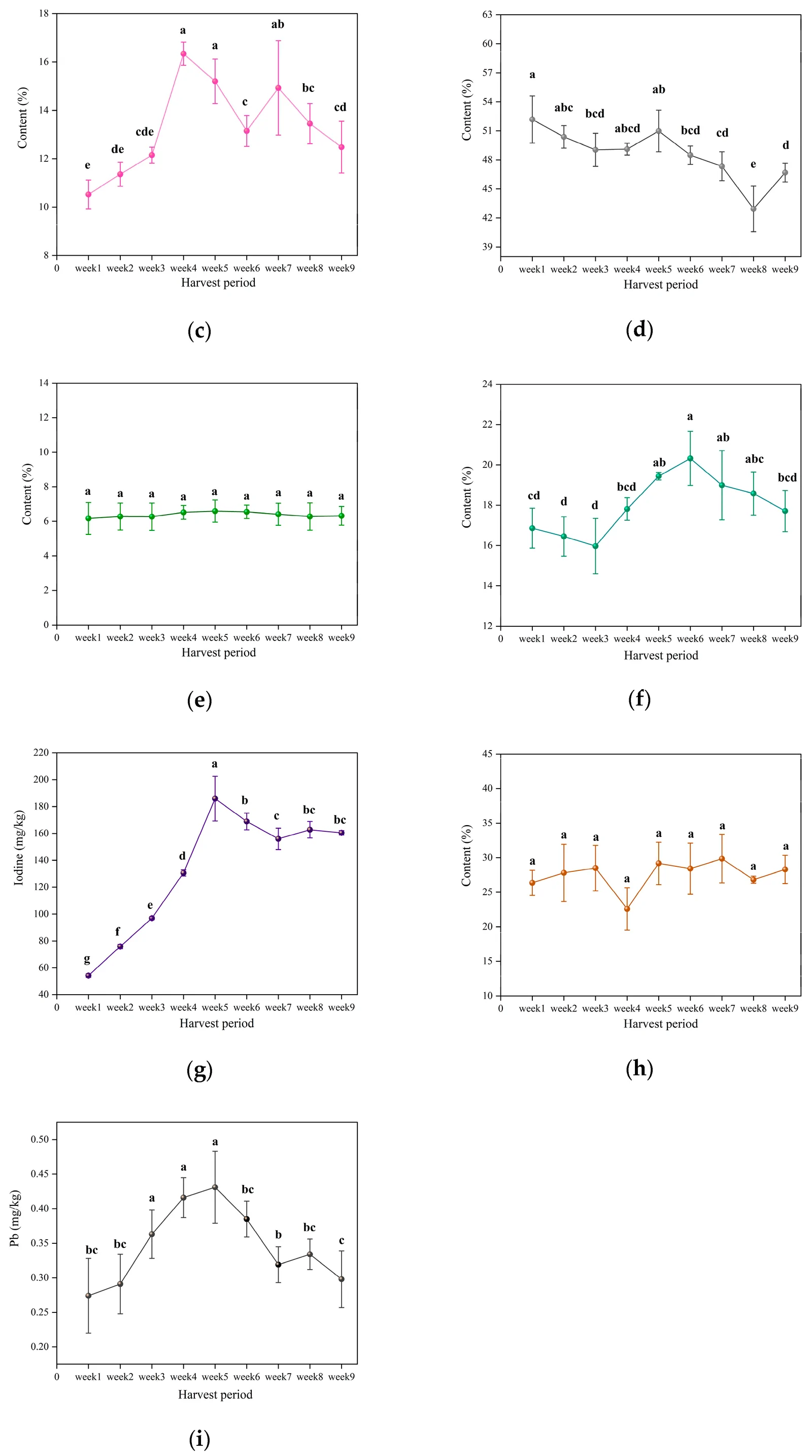
Changes in the major components of salted *U. pinnatifida* during the harvest period: (**a**) moisture; (**b**) fat; (**c**) protein; (**d**) ash; (**e**) mannitol; (**f**) alginate; (**g**) iodine; (**h**) fucoidan; and (**i**) Pb. All contents except moisture are expressed on a dry-weight basis. Letters (a, b, c, d, e, f, g) in the figure/table represent the results of multiple-comparison analysis while groups with completely different letters indicate significant differences at *p* < 0.05, and do not directly represent the magnitude of numerical values.

Salting produced distinct changes in the composition of *U. pinnatifida* (Figure 4). Fat content decreased slightly (Figure 4b), while protein content decreased by approximately 5% relative to blanched samples (Figure 4c). Ash content increased by approximately 10% (Figure 4d), reflecting substantial uptake of salt-derived minerals. Mannitol was extensively lost because of its high water solubility and stabilized at approximately 6% (Figure 4e). Alginate content decreased from approximately 30% in blanched samples to approximately 18% after salting (Figure 4f). Iodine content also decreased slightly (Figure 4g). The extraction yield of fucoidan sulfate was 0.65%, with a maximum total sugar content of 29.85%, considerably lower than in fresh or blanched samples (Figure 4h).

Pb content increased significantly during salting, reaching a maximum of 0.431 mg/kg and exceeding the levels in fresh samples (Figure 4i). Although blanching removed some Pb from fresh thalli, salting reintroduced Pb, presumably from the salt. Blanching denatures *U. pinnatifida* proteins and eliminates their enzymatic and physiological activities, but most amino acid side-chain functional groups remain intact. Protein unfolding can also expose additional sites for Pb^2+^ complexation. Alginate is a structural cell wall polysaccharide without intrinsic enzymatic activity. Short-term blanching does not degrade its polymer backbone or eliminate its abundant carboxylate adsorption sites. Blanching primarily disrupts cell membrane barriers, exposing intracellular and cell wall binding sites to Pb^2+^ in the salting brine. This further contributes to increased Pb content during salting. The edible salt contained 0.056 mg/kg Pb, complying with the relevant food safety limits. Nevertheless, *U. pinnatifida* may progressively accumulate Pb from the salting environment during the 48-h treatment because of its strong heavy metal adsorption capacity. This may increase the risk of excessive Pb levels in the final product.

### 3.5. Principal Component Analysis of Undaria pinnatifida Under Different Processing Treatments

**Figure 5:**
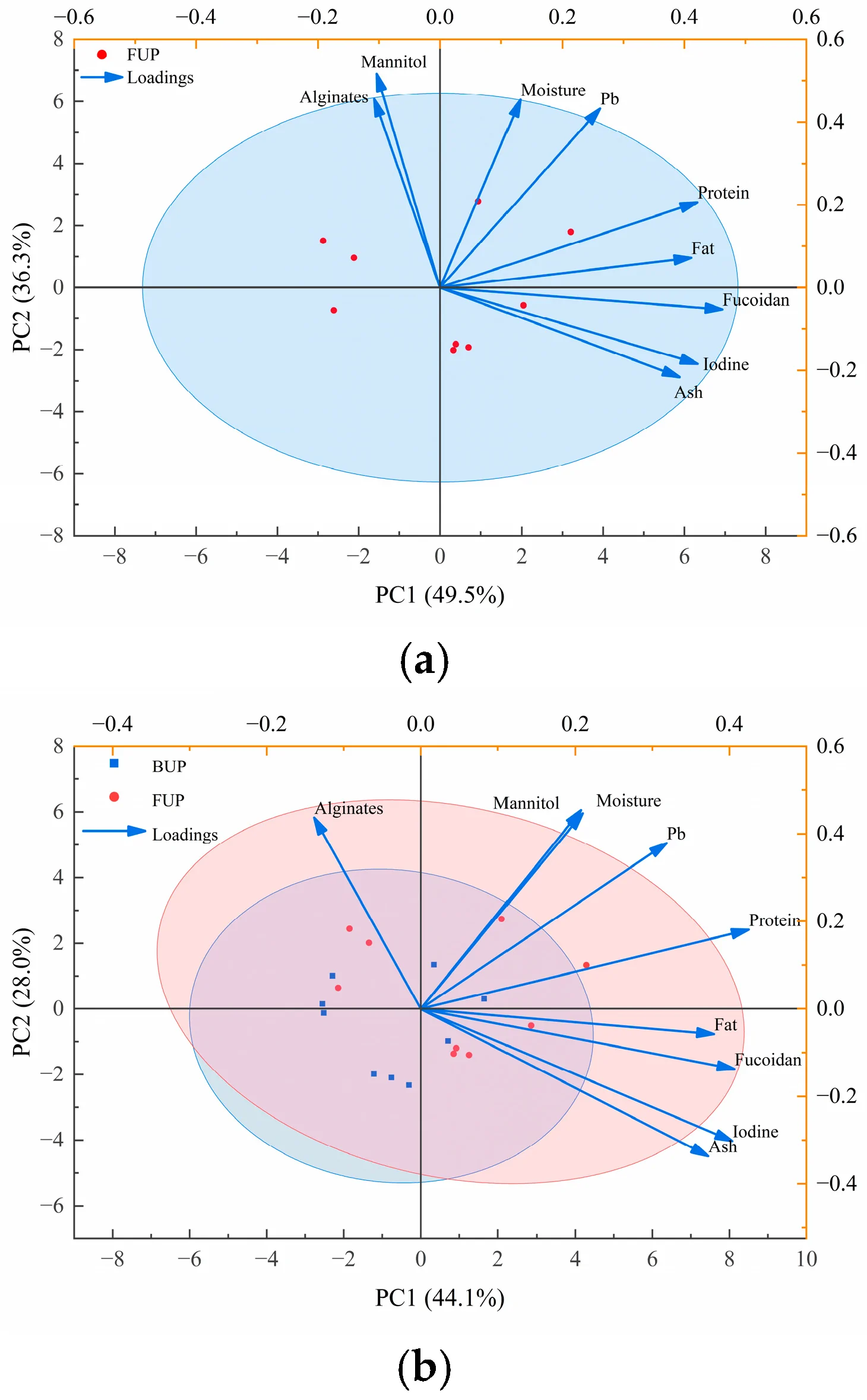

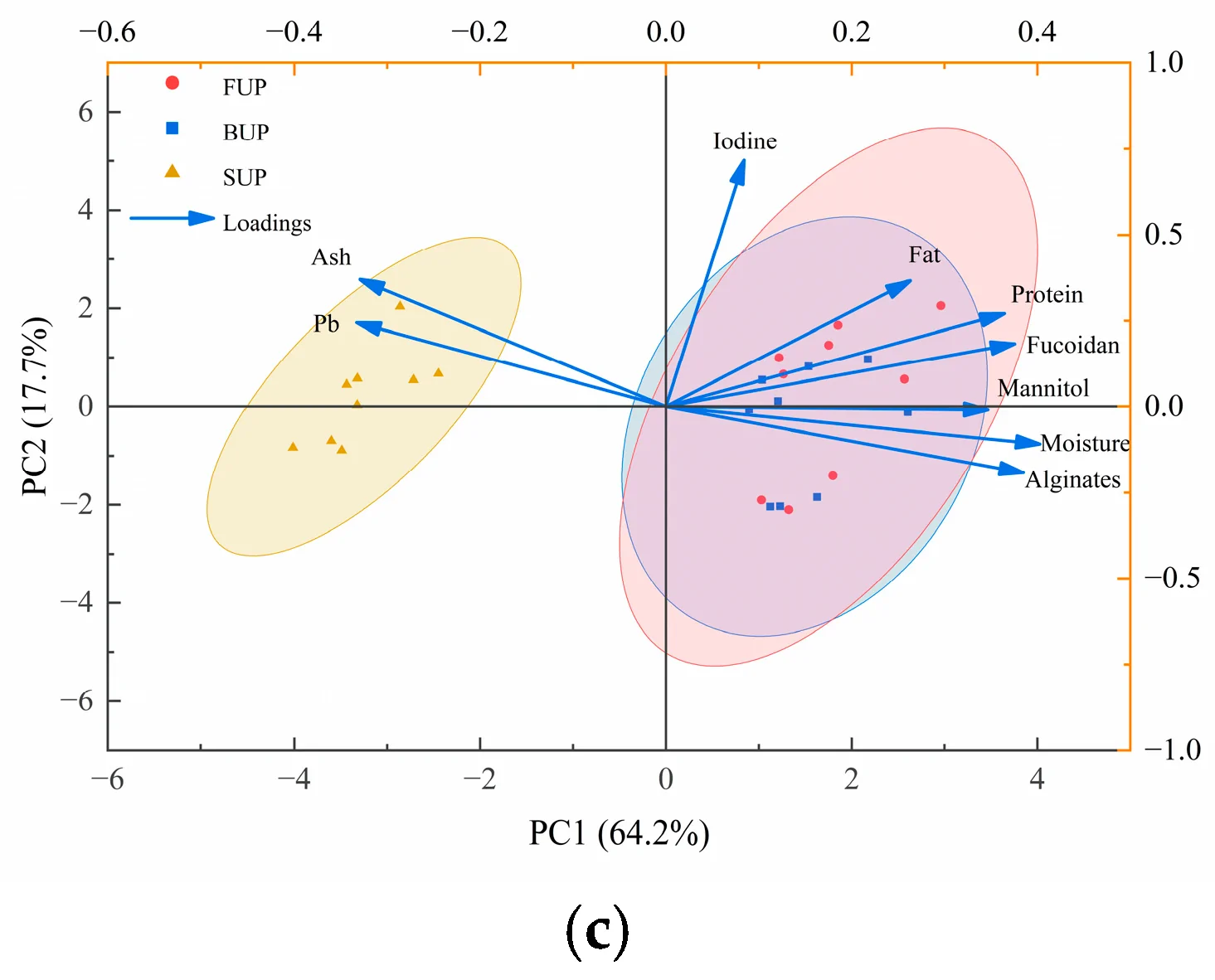
PCA biplots of physicochemical indices and Pb content in *U. pinnatifida* subjected to different processing treatments: (**a**) fresh samples (blue shaded ellipse); (**b**) fresh (red shaded ellipse) and blanched samples (blue shaded ellipse); and (**c**) fresh (red shaded ellipse), blanched (blue shaded ellipse), and salted samples (yellow shaded ellipse). The shaded ellipses represent 95% confidence ellipses for each group. Partial overlap of the ellipses indicates that some samples share similar chemical characteristics, while an overall separation trend among groups can still be observed.

Principal component analysis (PCA) was performed to visualize differences in physicochemical properties and Pb accumulation among samples subjected to different processing treatments (Figure 5).

Fresh samples clustered within a defined region with moderate within-group dispersion when analyzed separately (Figure 5a). Harvest time significantly affected nutritional composition and Pb accumulation. Pb was significantly and positively correlated with protein and alginate contents in fresh tissues. These macromolecules may therefore serve as important organic carriers involved in Pb binding and retention [33].

The PCA model incorporating fresh and blanched samples showed that blanching partially altered composition and Pb distribution (Figure 5b). Fresh and blanched samples formed distinguishable clusters in the two-dimensional principal component space. Blanching weakened the association between alginate and Pb accumulation, whereas the positive protein–Pb correlation remained largely intact. Protein may therefore have remained an important component involved in Pb binding. Blanched samples also showed clear losses of moisture and mannitol relative to fresh samples, consistent with the compositional changes described above [34].

The PCA model incorporating fresh, blanched, and salted samples identified salting as the main factor driving overall compositional differences (Figure 5c). The first principal component (PC1) explained 64.2% of the variance and completely separated salted samples from fresh and blanched samples. Fresh and blanched samples occupied the positive side of PC1, where protein, alginate, mannitol, and moisture were positively correlated. Pb accumulation occurred alongside these organic macromolecules. Salted samples occupied the negative side of PC1 and had markedly higher Pb, ash, and iodine contents. This separation suggests that salting may disrupt organic binding sites and alter relationships between Pb and components such as protein and alginate [35]. The nonoverlapping 95% confidence ellipses further confirmed significant differences in overall physicochemical characteristics among the three processing treatments.

### 3.6. Pearson Correlation Analysis of Components and Pb in Processed Undaria pinnatifida

**Figure 6:**
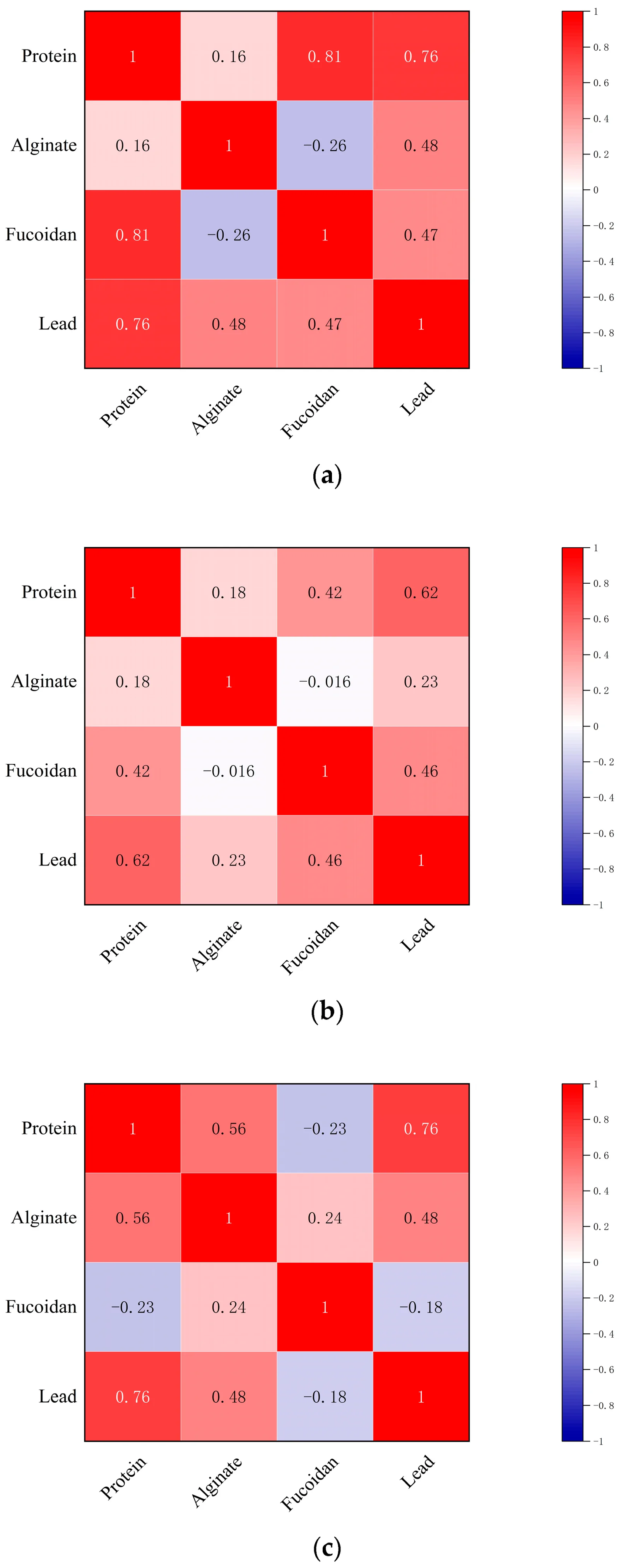
Pearson correlations between major components and Pb content in *U. pinnatifida*: (**a**) fresh; (**b**) blanched; and (**c**) salted samples.

Pearson correlation analysis was used to examine relationships between major components and Pb content under different processing conditions (Figure 6). In fresh samples, protein was strongly and positively correlated with Pb (r = 0.76, *p* < 0.05; Figure 6a). This suggests that proteins may play a dominant role in Pb binding in their native state. Alginate (r = 0.48, *p* < 0.05) and fucoidan (r = 0.47, *p* < 0.05) were also positively correlated with Pb content. These findings indicate that sulfate and carboxyl groups in these polysaccharides contribute to heavy metal adsorption [36].

After blanching, the protein–Pb correlation decreased slightly (r = 0.62, *p* < 0.05; Figure 6b), likely because of protein loss and heat-induced structural changes. The alginate–Pb correlation weakened considerably (r = 0.23, *p* < 0.05), possibly reflecting the removal of weakly bound Pb from cell walls. The fucoidan–Pb correlation (r = 0.46, *p* < 0.05) remained comparable to that in fresh samples, potentially reflecting fucoidan’s relatively high thermal stability under the blanching conditions.

Correlation patterns became more complex after salting (Figure 6c). Salting caused substantial losses of protein, alginate, and fucoidan, while high Na^+^ concentrations introduced strong ionic competition [37]. The protein–Pb correlation increased to r = 0.76 (*p* < 0.05), exceeding that in blanched samples. This may reflect enhanced protein binding to Pb in the Pb-containing salting medium. The alginate–Pb correlation remained unchanged (r = 0.48, *p* < 0.05). The fucoidan–Pb correlation shifted from positive to negative (r = −0.18, *p* < 0.05). This suggests that Na^+^ occupied fucoidan binding sites or that high salinity promoted fucoidan degradation, altering its interaction with Pb.

Overall, proteins and polysaccharides (alginate and fucoidan) were identified as the major Pb-binding components in fresh *U. pinnatifida*. Their contents showed relatively strong positive correlations with Pb content.

### 3.7. Distribution of Pb Chemical Forms in U. pinnatifida Under Different Processing Treatments

**Figure 7:**
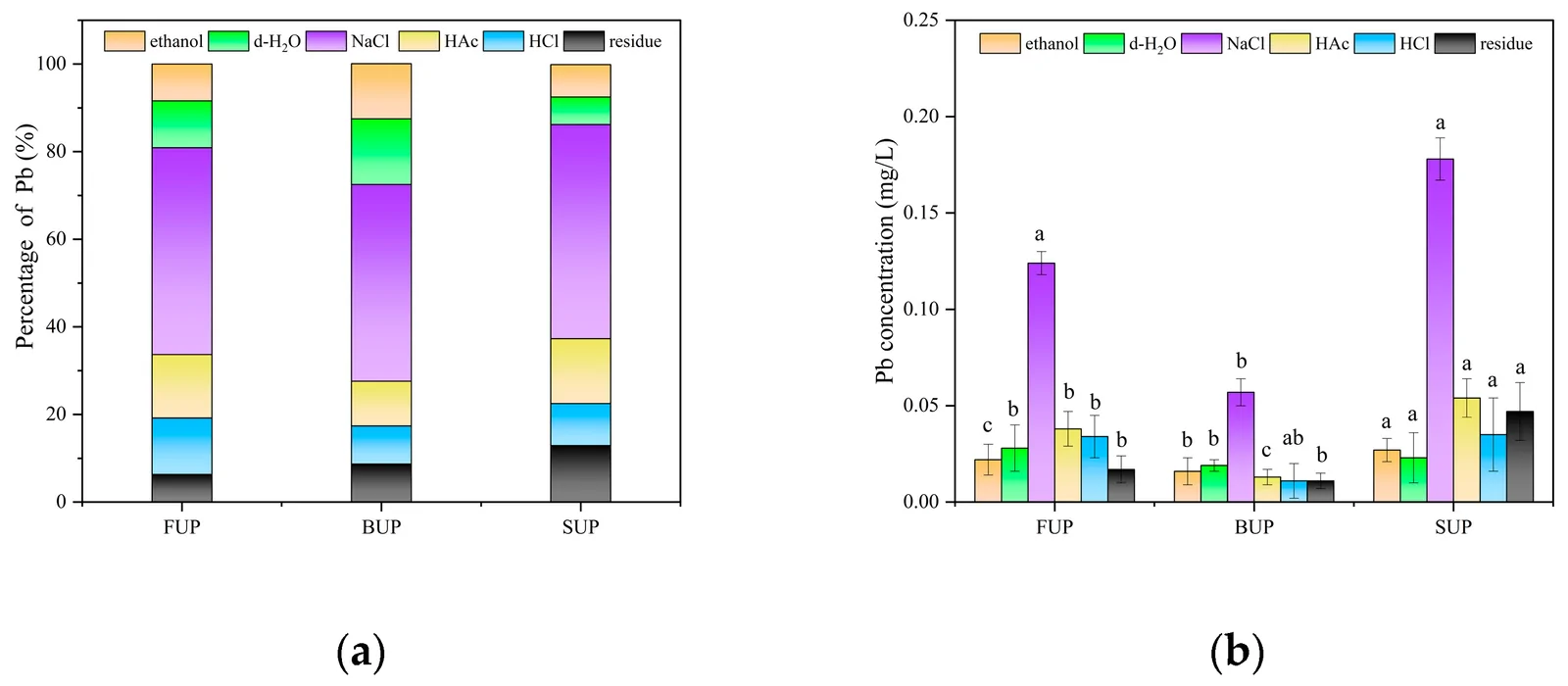
(**a**) Distribution of different chemical forms of Pb in fresh, blanched, and salted *U. pinnatifida*. (**b**) Different forms of Pb concentration in fresh, blanched, and salted *U. pinnatifida*. Different letters indicate statistically significant differences (*p* < 0.05).

The chemical form of a heavy metal is closely related to its bioavailability and toxicity [38]. Samples from the harvest batch with the highest Pb content were selected for chemical fractionation analysis. Total Pb contents in the corresponding fresh, blanched, and salted samples were 0.263, 0.127, and 0.364 mg/kg, respectively. The distribution of Pb forms is shown in Figure 7a. Of the six operationally defined fractions, the 1 mol/L NaCl-extractable fraction accounted for the largest proportion in all samples. This fraction, considered to represent Pb bound to proteins and polysaccharides, accounted for 27.87%, 32.70%, and 24.62% of Pb in fresh, blanched, and salted samples, respectively (Figure 7b).

The proportions of Pb in the other fractions were broadly similar in fresh samples. The proportions extracted by 80% ethanol (*v*/*v*) and deionized water were relatively low compared with blanched and salted samples. The residual fraction was smallest in blanched samples and largest in salted samples. These differences suggest that Pb in fresh tissues occurs predominantly in relatively stable bound forms [19]. Blanching partially disrupted cellular structure and released some weakly bound Pb, resulting in a relatively small residual fraction. Sheng likewise reported that thermal hydrolysis of sewage sludge reduced heavy metal concentrations and associated environmental risks [39].

During salting, trace Pb introduced from the processing environment, likely through the salt, may have been readsorbed by the algae. This increased total Pb content and appeared to stabilize its chemical forms, as indicated by the largest residual Pb fraction in salted samples. Although this stabilization may reduce Pb toxicity to some extent, elevated total Pb concentrations may still pose a food safety risk.

### 3.8. Regulation of Component Composition, Product Quality, and Pb Content Under Different Salting Conditions

#### 3.8.1. Changes in Component Composition

To examine how salting conditions affect Pb reduction, blanched *U. pinnatifida* from the batch with the highest Pb content (week 5) was treated with five salt concentrations (30–50%, *w*/*w*). Three salting durations (24, 36, and 48 h) were evaluated, and the resulting compositional changes were analyzed (Table 3).

Most component contents decreased relative to blanched samples under all salting conditions. At a given salt concentration, moisture content decreased with salting duration, reaching a minimum of approximately 60% in the 50% salt treatment. Iodine content also declined with salting duration, reaching its lowest value of 135.29 mg/kg at 50% salt. Fat, alginate, and mannitol contents did not change significantly across salting conditions.

Ash content increased with salt concentration and salting duration, reaching a maximum of approximately 50.16%. Protein content decreased slightly, approaching a minimum of 13.39%.

#### 3.8.2. Texture Profile Analysis

**Figure 8:**
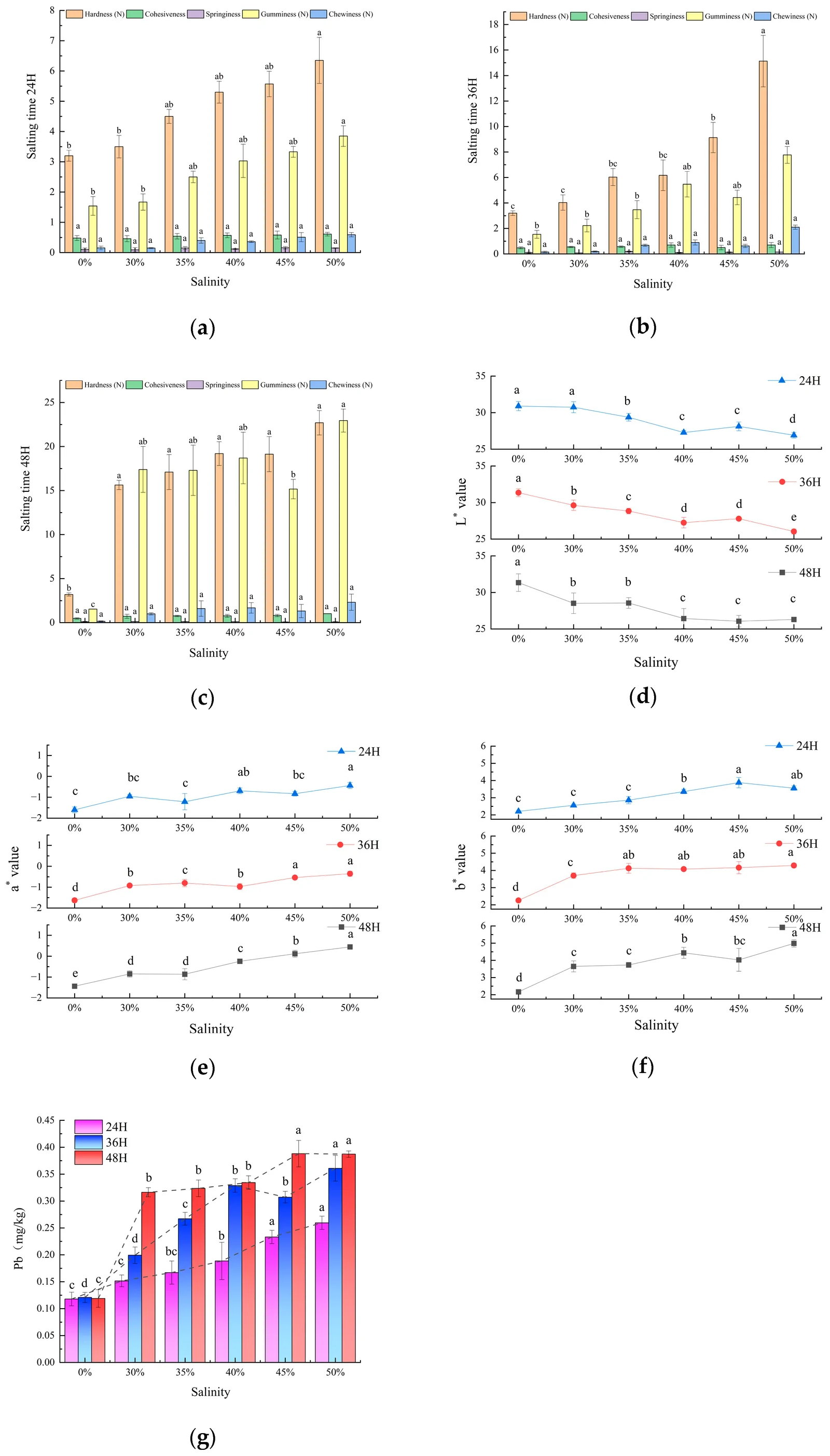
Effects of salting conditions on *U. pinnatifida*: (**a**–**c**) textural properties; (**d**–**f**) color; and (**g**) Pb content. Different letters indicate statistically significant differences (*p* < 0.05).

Hardness, springiness, and other textural parameters are important quality indicators reflecting the structure of salted *U. pinnatifida* [40]. Salt concentration was the main factor affecting texture in this experiment. At a given salting duration, hardness increased with salt concentration (Figure 8a–c). High salt concentrations create a strong osmotic gradient that promotes cellular water loss, reduces turgor, and induces tissue shrinkage. Salt penetration may also induce protein salting-out and denaturation and promote the formation of a denser alginate gel network, producing firmer tissues.

After 48 h, the 50% salt treatment produced the greatest hardness, considerably exceeding that after 24 h at the same salt concentration. Cohesiveness also increased with salt concentration and salting duration, indicating progressive tissue densification. Excessive hardness and cohesiveness may, however, impair mouthfeel.

Springiness was comparable across salt concentrations at 36 h but declined substantially in all treatments at 48 h. This decrease may reflect continuous osmotic dehydration, causing cell shrinkage, reduced tissue fullness, and losses of structural components such as alginate. Moderate hardness and relatively high springiness generally indicate a crisp, tender texture in salted *U. pinnatifida*. Overall, 35–40% salt for 48 h produced the most favorable textural properties.

#### 3.8.3. Color Analysis

Color is a key quality attribute of salted *U. pinnatifida* that directly affects consumer perception and acceptance. Marianela reported that mature *U. pinnatifida* naturally contains higher pigment levels and exhibits more favorable color parameters [41].

L* values (lightness) decreased with salting duration in all treatments (Figure 8d). This indicates less pronounced chlorophyll-related color deterioration at lower salt concentrations, whereas higher concentrations caused tissues to darken and lose luster. Endo similarly reported that suitable nutrient availability is required to maintain high pigment levels in *U. pinnatifida* [42].

a* values (redness/greenness) increased with salt concentration at all salting durations, and values at 48 h significantly exceeded those at shorter durations (Figure 8e). The pronounced increase at 50% salt indicated duller green coloration and substantial loss of the original bright-green hue. This was likely caused by pigment degradation or structural changes induced by osmotic stress.

b* values (yellowness/blueness) also increased with salt concentration (Figure 8f). At 48 h, the 40% and 45% salt treatments had significantly lower b* values than the other treatments. These samples therefore retained a color closer to the original green appearance and showed no obvious yellowing.

#### 3.8.4. Changes in Pb Content

Changes in Pb content under different salting conditions are shown in Figure 8g. At 24 h, Pb content increased with salt concentration, from 0.15 to 0.26 mg/kg. At 36 h, Pb content tended to stabilize in the 40–50% salt treatments, reaching a maximum of 0.36 mg/kg. At 48 h, the highest Pb content (0.39 mg/kg) occurred at 50% salt. Thus, salting with salt that met the Pb limit still resulted in relatively elevated Pb levels in algal tissues. Since the blanching pretreatment induced cell death and abolished metabolic activity of *U. pinnatifida*, active biological uptake of Pb does not occur during salting. The redistribution of lead is governed by passive diffusion of lead ions, together with adsorption and binding reactions with organic components of seaweed tissue. Lower salt concentrations and shorter salting durations reduced Pb accumulation, but suitable processing conditions must also preserve product quality and appearance [43].

Salting with 40% salt for 48 h was selected based on a combined assessment of nutritional composition, texture, color, and residual Pb concentration. Salting reduced moisture, increased ash, and caused partial losses of iodine and protein, whereas alginate, mannitol, and fat remained relatively stable. Excessive salting at 50% increased nutrient depletion and produced overly hard tissues, pronounced green fading, yellowing, and the highest Pb concentration (0.39 mg/kg). Lower-salt treatments produced lower Pb residues but did not achieve the required product quality. The 40% salt treatment for 48 h retained nutritional components, maintained moderate hardness and good springiness, preserved green coloration, and kept Pb below 0.35 mg/kg. This condition balances nutrient retention, sensory acceptability, and heavy metal safety in salted *U. pinnatifida.* Because Codex Alimentarius has not established a specific maximum Pb limit for edible seaweed or wakame, the product was assessed against GB 2762-2025 [44]. This National Food Safety Standard—Limits of Contaminants in Food specifies a maximum Pb level of 1.0 mg/kg for algae products, excluding spirulina products, on the basis of the usually edible portion. The Pb level obtained under the selected salting condition complied with this limit.

#### 3.8.5. Health Risk Assessment of Pb

Pb health risks in optimized salted *Undaria pinnatifida* were assessed for children, adolescents, and adults using daily intake of metals (DIM), hazard quotient (HQ), cancer risk (CR), and total cancer risk (TCR). DIM decreased with age in the order children > adolescents > adults. HQ values were well below 1 in all groups, and CR values were below 10^−6^, suggesting acceptable noncarcinogenic risk and negligible carcinogenic risk [45]. Under the joint constraints of HQ ≤ 1 and CR ≤ 10^−6^, following the USEPA lifetime exposure principle, the estimated maximum permissible daily intakes were approximately 6.7 g/d (DW) for children, 13.4 g/d (DW) for adolescents, and 20.2 g/d (DW) for adults [46]. Carcinogenic risk constrained the intake limit in every population group. The final product’s Pb content met the Codex Alimentarius standard. These intake recommendations provide practical guidance for consuming salted seaweed, considering Pb toxicity to multiple organs and the high frequency of seaweed consumption in Asian populations.

A maximum permissible intake was proposed despite the negligible estimated carcinogenic risk (CR < 10^−6^), because negligible risk does not imply zero risk. This limit provides a safety margin for variation in heavy metal concentrations and individual consumption patterns, offering practical dietary guidance.

## 4. Conclusions

This study characterized changes in nutritional components, bioactive constituents, and Pb content in fresh *U. pinnatifida* and its blanched and salted products throughout the harvest period. Alginate, protein, iodine, and other major components showed distinct seasonal patterns, while blanching and salting affected algal composition differently. Pb concentration was positively associated with protein and alginate contents, suggesting that these components may provide important Pb-binding sites. Blanching reduced Pb levels, whereas salting promoted Pb accumulation in the final product. These findings highlight the importance of controlling potential contaminant inputs during postharvest processing.

Salting with 40% salt for 48 h provided a favorable balance of texture, color, and Pb control, although residual Pb remained detectable. Chemical fractionation indicated that Pb in fresh *U. pinnatifida* occurred predominantly in relatively stable bound forms. Blanching increased Pb mobility by releasing weakly bound fractions, whereas subsequent salting shifted its distribution toward more stable fractions. Reduced Pb extractability does not indicate complete detoxification. Sequential extraction provides an approximate assessment of Pb fraction distribution. Furthermore, the continuous extraction method has limitations in the determination of lead speciation distribution, and the potential food safety risk requires further evaluation.

Future research should clarify Pb enrichment and tissue-level distribution in freshly harvested *U. pinnatifida* and the mechanisms governing Pb adsorption and translocation under natural marine conditions. Rapid, sensitive methods are also needed to monitor Pb throughout aquatic food processing. Scalable Pb-removal and risk-mitigation technologies compatible with industrial processing should be developed and validated. These efforts would support safer processing and the sustainable, high-quality utilization of marine algae from ocean to table.

## Figures and Tables

**Table 1:** Microwave digestion conditions for *U. pinnatifida.*

Step	Temperature (°C)	Pressure (atm)	Duration (min)
1	80	10	3
2	120	20	3
3	150	30	3
4	180	40	10

**Table 2:** Variation in the physical characteristics of *U. pinnatifida* during the harvesting period.

Harvest Week	FSWP (%)	BSWP (%)	FL (cm)	FW (cm)	BL (cm)	BW (cm)
Week 1	33.76 ± 1.49 ^b^	41.00 ± 4.02 ^abc^	132.00 ± 8.00 ^b^	36.00 ± 6.25 ^b^	126.00 ± 3.61 ^d^	35.00 ± 1.73 ^bc^
Week 2	40.64 ± 4.13 ^ab^	32.45 ± 2.87 ^c^	141.00 ± 1.73 ^a^	38.00 ± 2.08 ^ab^	130.00 ± 4.16 ^cd^	32.00 ± 2.65 ^c^
Week 3	40.49 ± 1.02 ^ab^	44.62 ± 1.86 ^ab^	143.00 ± 4.36 ^a^	40.00 ± 2.65 ^ab^	143.00 ± 4.36 ^a^	32.00 ± 2.36 ^c^
Week 4	35.47 ± 0.26 ^ab^	40.08 ± 4.63 ^abc^	147.00 ± 9.71 ^a^	46.00 ± 4.58 ^a^	139.00 ± 6.00 ^ab^	35.00 ± 2.00 ^bc^
Week 5	42.41 ± 9.52 ^ab^	49.19 ± 12.15 ^a^	147.00 ± 1.53 ^a^	45.00 ± 3.06 ^a^	138.00 ± 2.08 ^abc^	34.00 ± 1.53 ^bc^
Week 6	40.40 ± 7.80 ^ab^	35.56 ± 7.12 ^bc^	144.00 ± 1.53 ^a^	41.00 ± 6.43 ^ab^	140.00 ± 2.52 ^ab^	35.00 ± 3.21 ^bc^
Week 7	39.48 ± 1.38 ^ab^	40.21 ± 3.43 ^abc^	146.00 ± 3.00 ^a^	40.00 ± 4.00 ^ab^	134.00 ± 3.00 ^abcd^	37.00 ± 2.08 ^ab^
Week 8	41.89 ± 1.64 ^ab^	41.95 ± 3.38 ^abc^	144.00 ± 5.00 ^a^	41.00 ± 2.65 ^ab^	129.00 ± 8.00 ^cd^	41.00 ± 2.65 ^a^
Week 9	43.99 ± 2.24 ^a^	43.39 ± 0.90 ^ab^	146.00 ± 2.65 ^a^	42.00 ± 4.00 ^ab^	131.00 ± 7.00 ^bcd^	42.00 ± 4.00 ^a^

FSWP, fresh *U. pinnatifida* stipe weight proportion; BSWP, blanched *U. pinnatifida* stipe weight proportion; FL, fresh thallus length; FW, fresh thallus width; BL, blanched thallus length; BW, blanched thallus width. Letters (a, b, c, d) in the figure/table represent the results of multiple-comparison analysis while groups with completely different letters indicate significant differences at *p* < 0.05, and do not directly represent the magnitude of numerical values; *n* ≥ 30.

**Table 3:** Changes in *U. pinnatifida* composition under different salting conditions.

Salting Time (h)	Salt Concentration (%)	Moisture (%)	Ash (%)	Fat (%)	Protein (%)	Mannitol (%)	Alginate (%)	Iodine (mg/kg)
0	0	90.31 ± 0.56 ^a^	39.75 ± 1.63 ^a^	0.27 ± 0.07 ^a^	19.95 ± 2.55 ^a^	15.42 ± 2.15 ^a^	38.05 ± 1.98 ^a^	198.36 ± 23.21 ^a^
24	30	69.67 ± 1.94 ^b^	45.87 ± 3.23 ^b^	0.21 ± 0.01 ^a^	14.87 ± 0.88 ^a^	6.84 ± 0.65 ^b^	19.35 ± 2.51 ^b^	168.36 ± 12.15 ^a^
	35	67.98 ± 3.06 ^b^	45.64 ± 2.23 ^b^	0.21 ± 0.01 ^a^	14.72 ± 1.13 ^a^	6.76 ± 0.71 ^b^	19.43 ± 1.84 ^b^	162.81 ± 7.36 ^a^
	40	68.03 ± 2.64 ^b^	47.86 ± 2.09 ^b^	0.22 ± 0.01 ^a^	14.38 ± 1.45 ^a^	6.81 ± 0.70 ^b^	19.26 ± 1.64 ^b^	166.03 ± 9.97 ^a^
	45	61.43 ± 0.96 ^c^	45.81 ± 3.27 ^b^	0.22 ± 0.02 ^a^	14.06 ± 1.52 ^a^	6.77 ± 0.73 ^b^	19.37 ± 1.91 ^b^	163.62 ± 5.52 ^a^
	50	60.99 ± 4.03 ^c^	48.92 ± 2.16 ^b^	0.18 ± 0.02 ^a^	14.11 ± 1.63 ^a^	6.71 ± 0.97 ^b^	19.45 ± 1.49 ^b^	157.73 ± 9.04 ^a^
36	30	66.34 ± 2.36 ^b^	47.21 ± 2.31 ^b^	0.20 ± 0.01 ^a^	14.71 ± 1.69 ^a^	6.79 ± 0.27 ^b^	19.47 ± 1.97 ^b^	149.18 ± 7.75 ^a^
	35	66.27 ± 1.46 ^b^	46.95 ± 1.64 ^b^	0.20 ± 0.02 ^a^	14.77 ± 1.06 ^a^	6.65 ± 0.68 ^b^	19.32 ± 2.13 ^b^	146.23 ± 8.82 ^a^
	40	65.74 ± 3.61 ^b^	48.34 ± 1.45 ^b^	0.20 ± 0.01 ^a^	14.05 ± 0.98 ^a^	6.55 ± 0.98 ^b^	19.33 ± 1.76 ^b^	147.33 ± 7.67 ^a^
	45	60.65 ± 1.76 ^c^	49.42 ± 2.64 ^b^	0.19 ± 0.03 ^a^	13.63 ± 0.86 ^a^	6.69 ± 0.48 ^b^	19.26 ± 1.73 ^b^	143.46 ± 7.07 ^a^
	50	59.67 ± 3.96 ^c^	50.16 ± 4.15 ^b^	0.20 ± 0.03 ^a^	13.79 ± 1.26 ^a^	6.62 ± 0.77 ^b^	19.28 ± 1.36 ^b^	144.07 ± 2.99 ^a^
48	30	63.09 ± 1.68 ^b^	46.89 ± 2.11 ^b^	0.20 ± 0.02 ^a^	14.68 ± 1.52 ^a^	6.15 ± 0.59 ^b^	18.79 ± 2.07 ^b^	151.45 ± 4.98 ^a^
	35	62.81 ± 2.11 ^b^	47.59 ± 3.87 ^b^	0.19 ± 0.02 ^a^	14.19 ± 1.71 ^a^	6.06 ± 0.46 ^b^	18.89 ± 1.91 ^b^	139.26 ± 10.11 ^a^
	40	61.53 ± 2.26 ^b^	47.66 ± 3.72 ^b^	0.18 ± 0.02 ^a^	13.64 ± 1.37 ^a^	5.99 ± 0.88 ^b^	19.06 ± 1.29 ^b^	148.32 ± 10.21 ^a^
	45	61.88 ± 3.12 ^b^	49.38 ± 1.57 ^b^	0.20 ± 0.01 ^a^	13.39 ± 1.66 ^a^	6.26 ± 0.35 ^b^	18.79 ± 1.92 ^b^	137.65 ± 8.92 ^a^
	50	60.75 ± 2.09 ^c^	49.14 ± 1.72 ^b^	0.19 ± 0.03 ^a^	13.65 ± 0.92 ^a^	5.92 ± 0.33 ^b^	18.63 ± 1.53 ^b^	135.29 ± 4.96 ^a^

Different letters indicate statistically significant differences (*p* < 0.05).

## Data Availability

The raw data supporting the conclusions of this study are available from the authors upon request.

## Appendix A

**Table A1:** Harvest dates and sample categories.

Harvest Date	Fresh *U. pinnatifida*	Blanched *U. pinnatifida*	Salted *U. pinnatifida*
25 February(week 1)	≥30	≥30	—
6 March(week 2)	≥30	≥30	≥30
13 March (week 3)	≥30	≥30	≥30
20 March (week 4)	≥30	≥30	≥30
27 March (week 5)	≥30	≥30	≥30
8 April (week 6)	≥30	≥30	≥30
15 April (week 7)	≥30	≥30	≥30
22 April (week 8)	≥30	≥30	≥30
29 April (week 9)	≥30	≥30	≥30
6 May	—	—	≥30

“—”, this symbol indicates that such samples have not been obtained.
